# 

**Published:** 1887-08-20

**Authors:** 


### CONTENTS.

**"Well-guessed" or "Well-done"?** - - - 341
**Words of Consolation.**—XLVI. The Sufferings of Christ - - - - - - - 342
**Poetry.**—An Army of Women - - - - 342
**The Doctor's Pulpit.**—Blood-letting - - - 343
**Religio Medici** - - - - - - 344
**The Story of the Hospitals.**—The Royal National Hospital for Consumption, Ventnor - - - 345
**The National Pension Fund** - - - - 347
**Notes and News.**— Miscellaneous Items. — Private Nursing Engagements.—Flower-shows, Bazaars, etc., in Aid of Hospitals.—Special Hospitals.—The Hartlepools Hospital.—Vacancies.—The Casualties at Whiteley's.—The Hospitals Association Meetings.—Nurses and their Work.—The Great Northern Central Hospital.—Annual Meetings and Reports.—The Norfolk and Norwich Hospital.—The Falkirk Association of Trained Nurses.—The Sandygate Sanatorium, etc. - - - 348
**Annotations.**—A Deluge of Doctors.—Men must do Something.—Why don't Doctors Emigrate?—A Short Way with Medical Aspirants - - - 351
**Chats about Medical Museums.**—By One of the Crowd.—IX. The Middlesex Hospital - - - 352
**Amusements for Convalescents.** — Palmistry Notes. By a Lady - - - - - 353
**The Origin of Hospitals** - - - - 354
**Incidents of Animal Life.**—About Birds - - 355
**The Editor's Letter-Box.**—A Heroic Fireman.—The Condition of Midwives - - - - - 356
**Questions and Answers** - - - - 356
**The Children's Corner.**—Puzzles, Answers, etc. - vi
**Amusements with Profit** - - - - vi

